# Supplementary figures and images for: Complementary performances of convolutional and capsule neural networks on classifying microfluidic images of dividing yeast cells
Source: PLoS One. 2021 Mar 17;16(3):e0246988. doi: 10.1371/journal.pone.0246988 (PMC7968698; doi:10.1371/journal.pone.0246988)

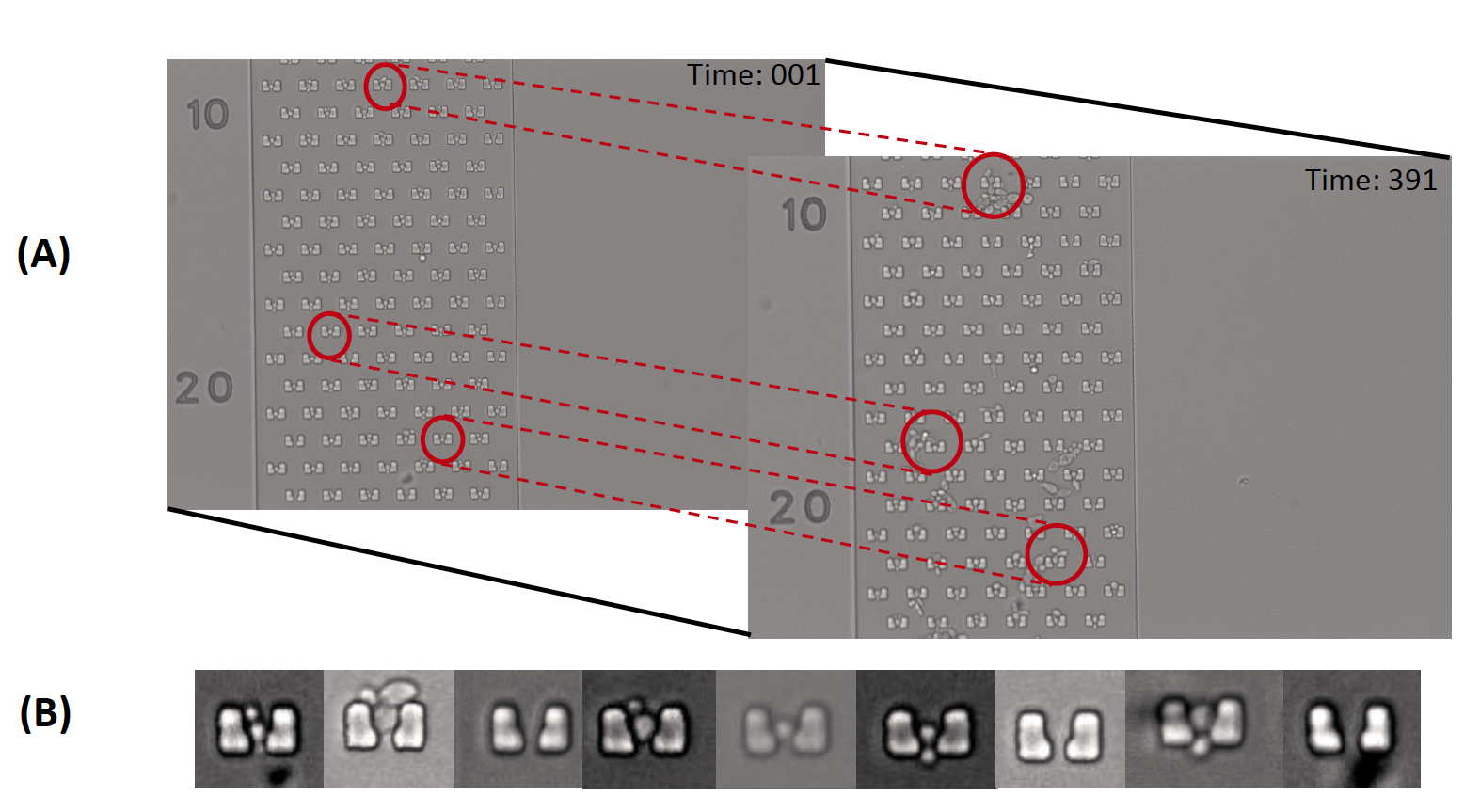

Supplement: S1 Fig — (A) Time-lapsed images from time-point 001 to time-point 391. Black circles with connected dash-lines indicate that some of the traps become overcrowded over time. (B) Each image partitioned to 60x60 pixels sub-images, and individual trap image is highly variable. While traps and cells have a limited number of orientations, the contrast, brightness, and image quality all add great complexity to the dataset. There are often shadows, depending on the lighting conditions of the experiment. (TIF) [file pone.0246988.s001.tif]

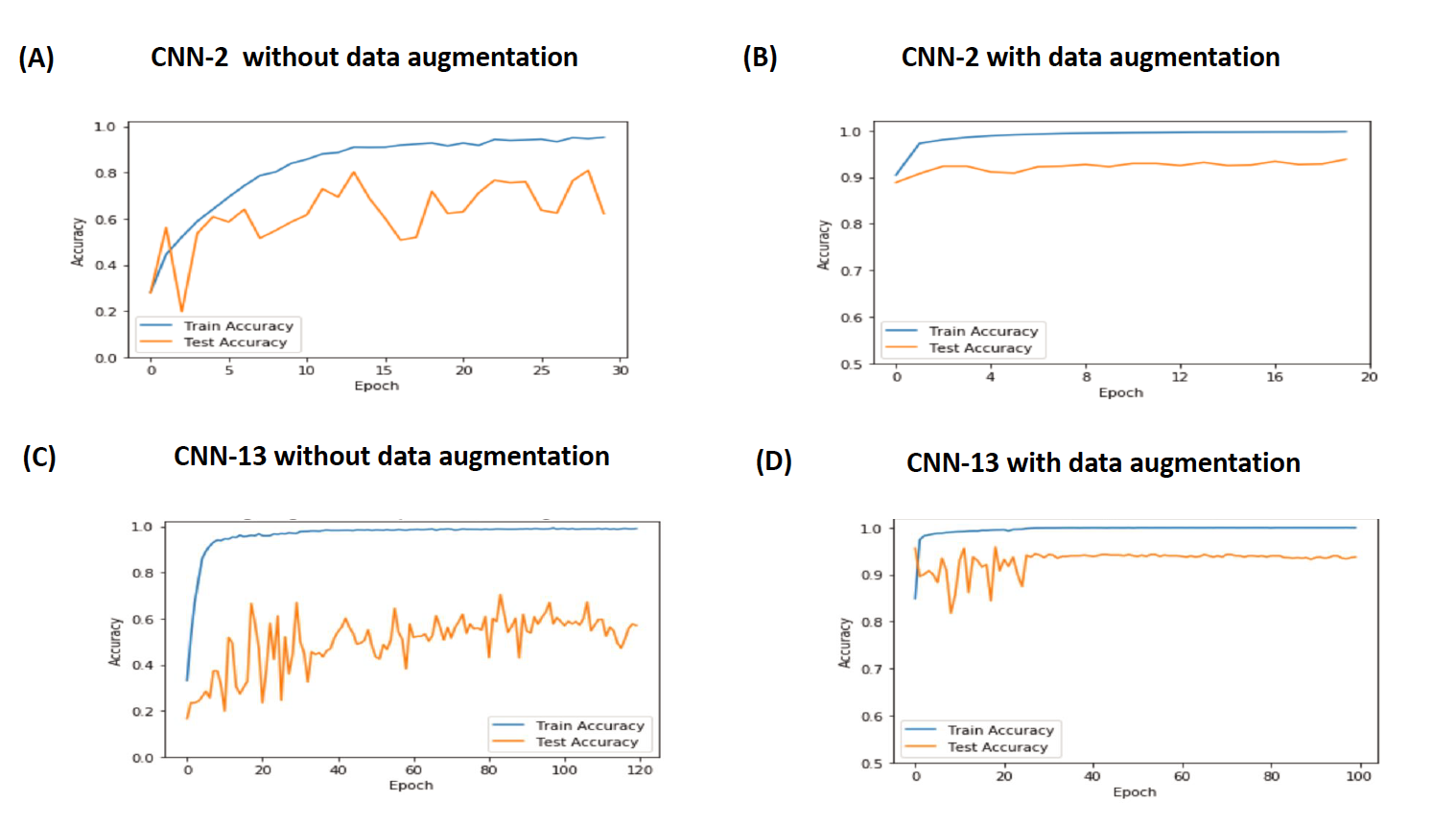

Supplement: S2 Fig — (A) and (B) are plots for the CNN-2 model without and with data augmentation. (C) and (D) are plots for CNN-13 model without and with data augmentation. (TIF) [file pone.0246988.s002.tif]

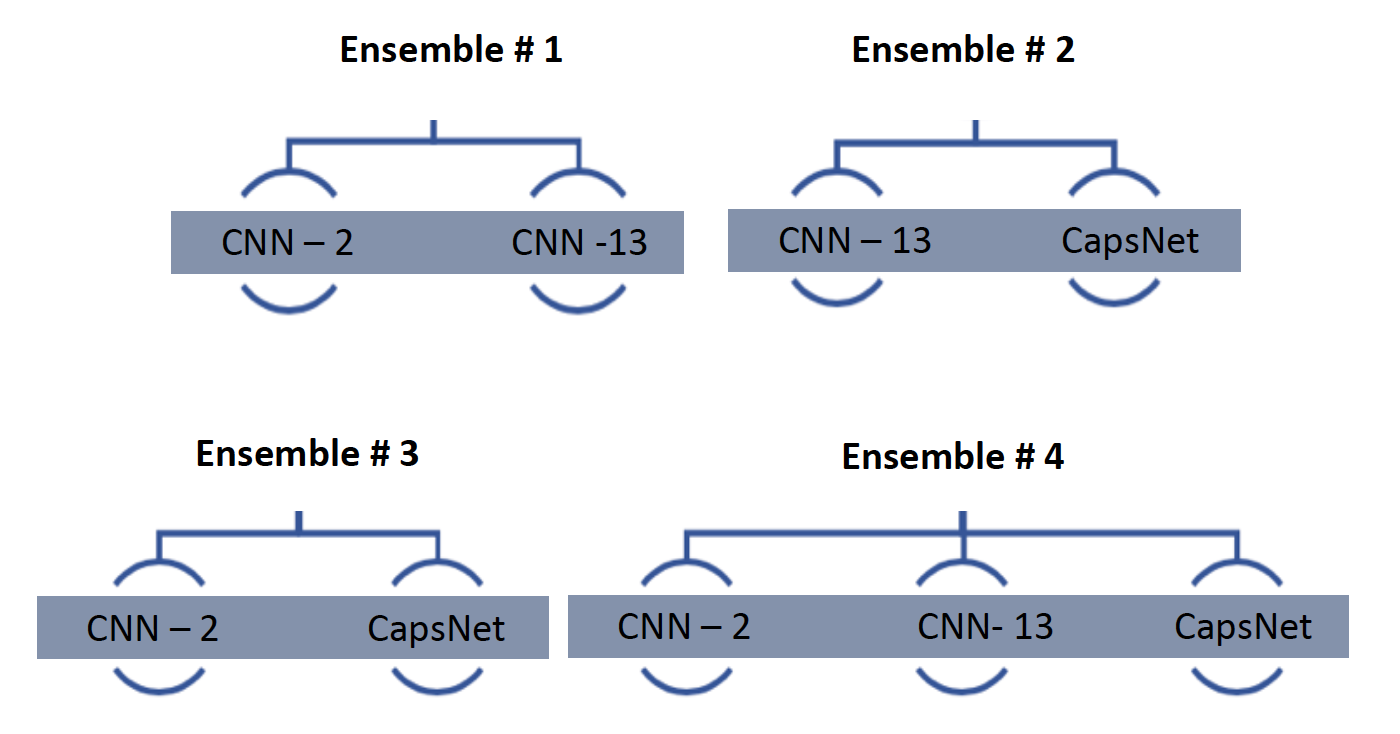

Supplement: S3 Fig — Results of CNN-2, CNN-13, and CapsNet models indicated that there are numerous ways to ensemble (i.e., combine) models together to create a single aggregate model. We explored the results from all possible ensembles with different combinations based on practical and key performance metrics. (TIF) [file pone.0246988.s003.tif]

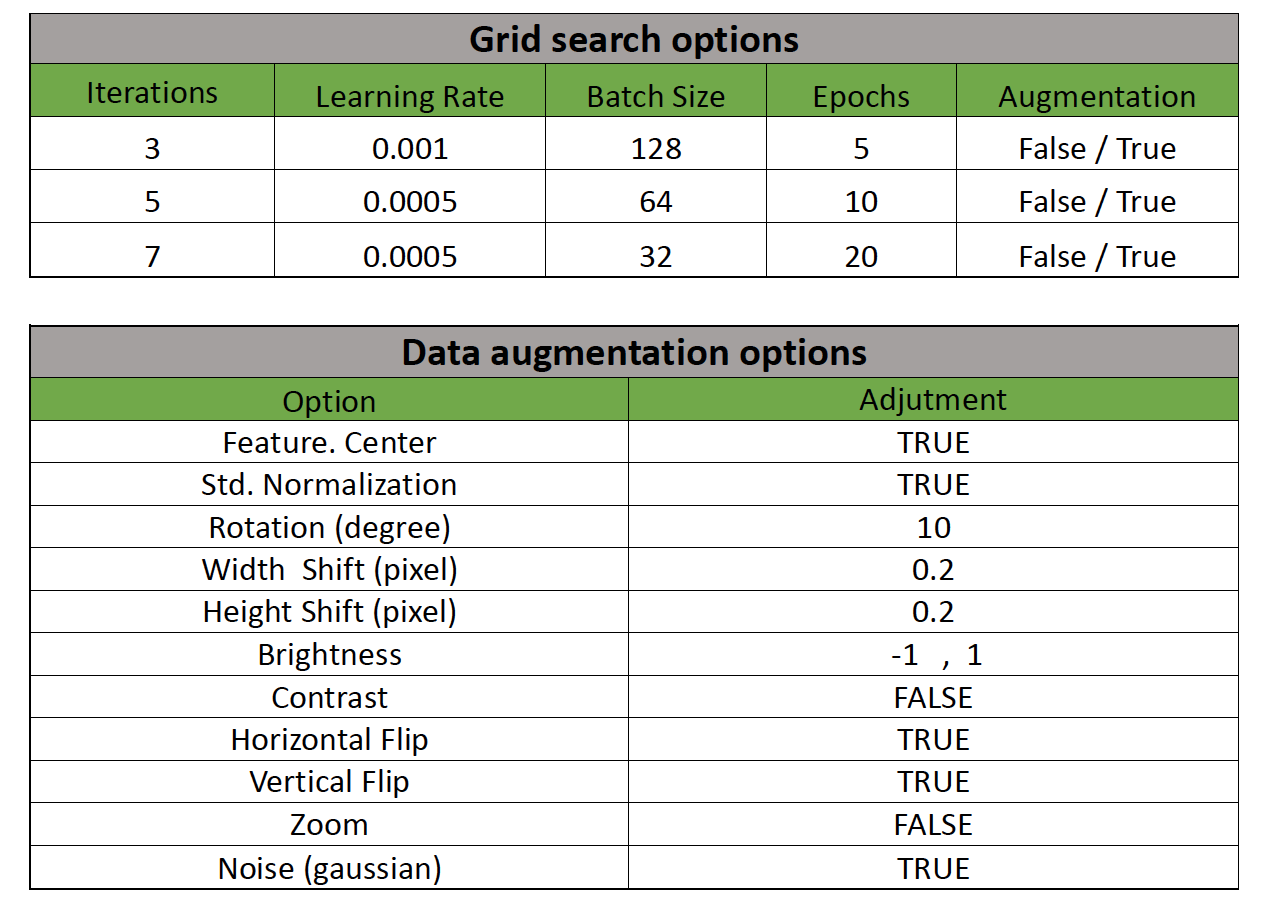

Supplement: S1 Table — The grid search option table used for all models and data augmentation features applied when the data augmentation in grid search option was set to “True.” (TIF) [file pone.0246988.s004.tif]

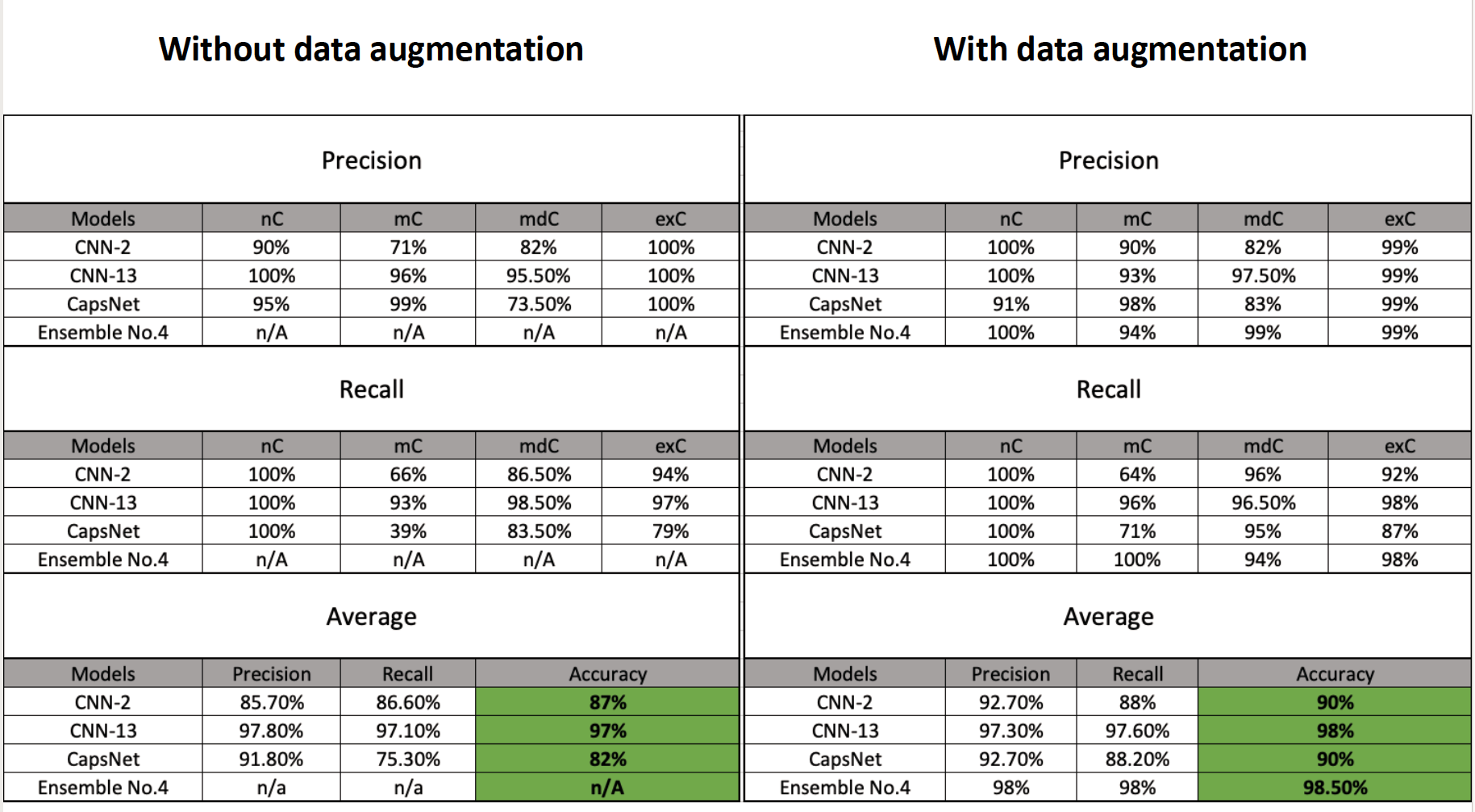

Supplement: S2 Table — The results of each model for individual biological class with effect of data augmentation. (TIF) [file pone.0246988.s005.tif]

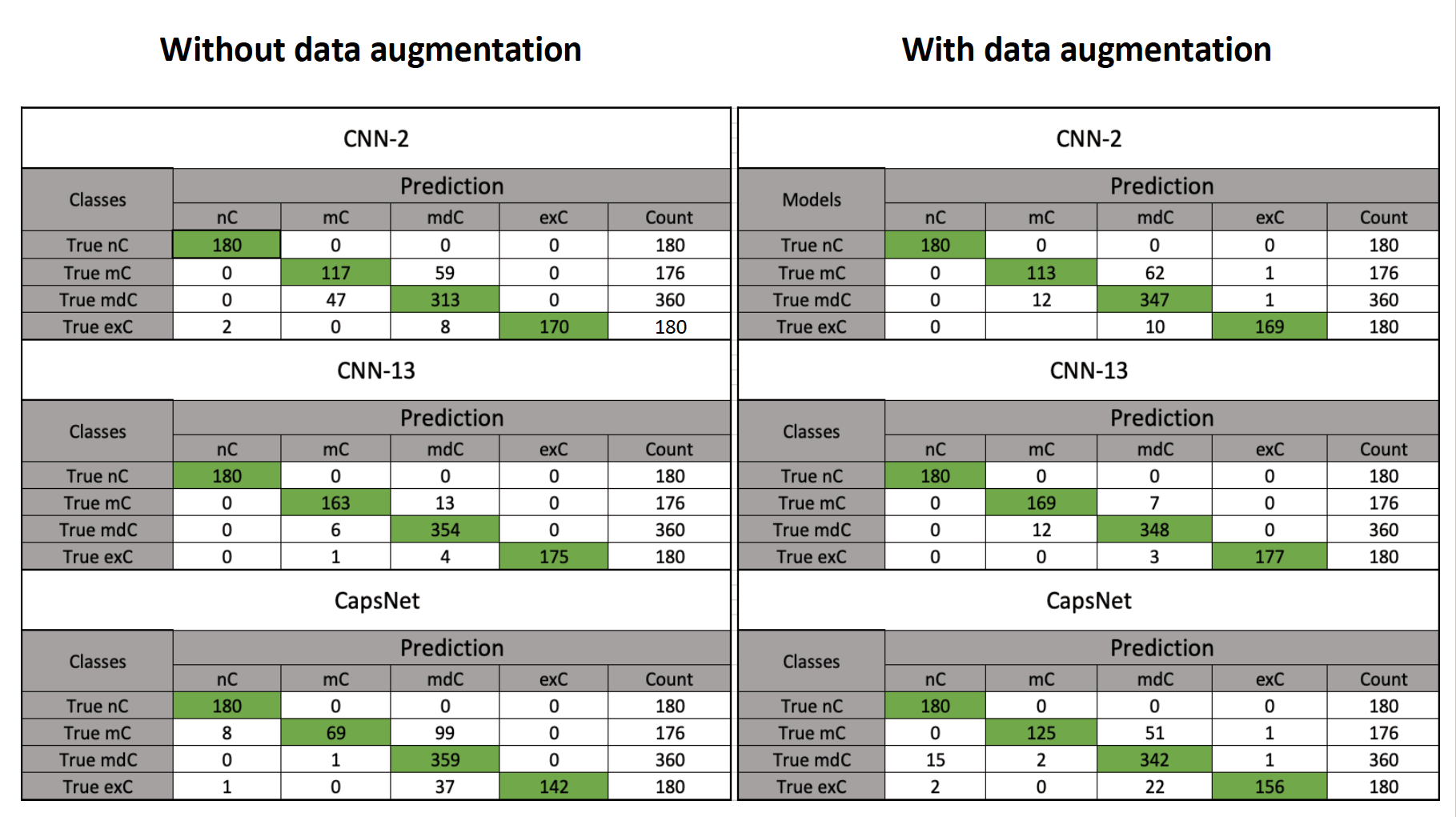

Supplement: S3 Table — Three models confusion matrix with indication of data augmentation effectiveness. (TIF) [file pone.0246988.s006.tif]

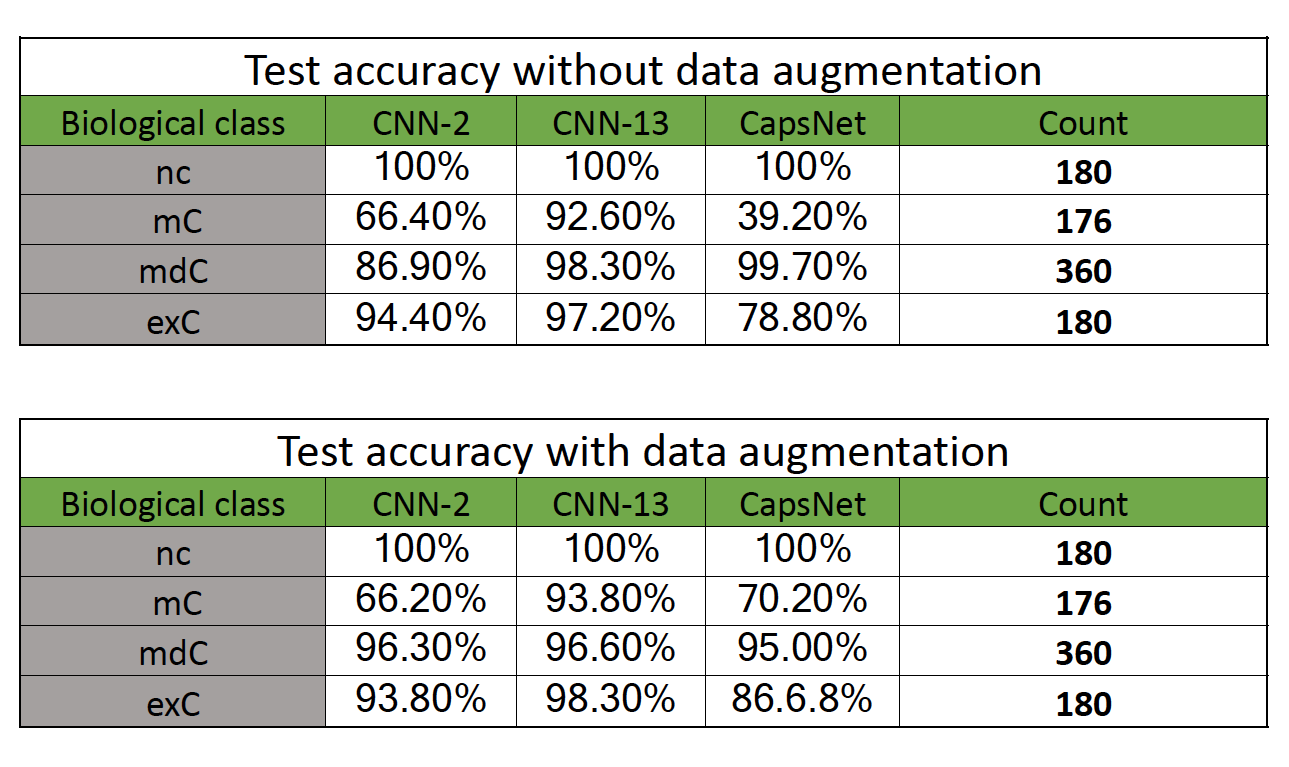

Supplement: S4 Table — Accuracy of models for individual biological class. (TIF) [file pone.0246988.s007.tif]

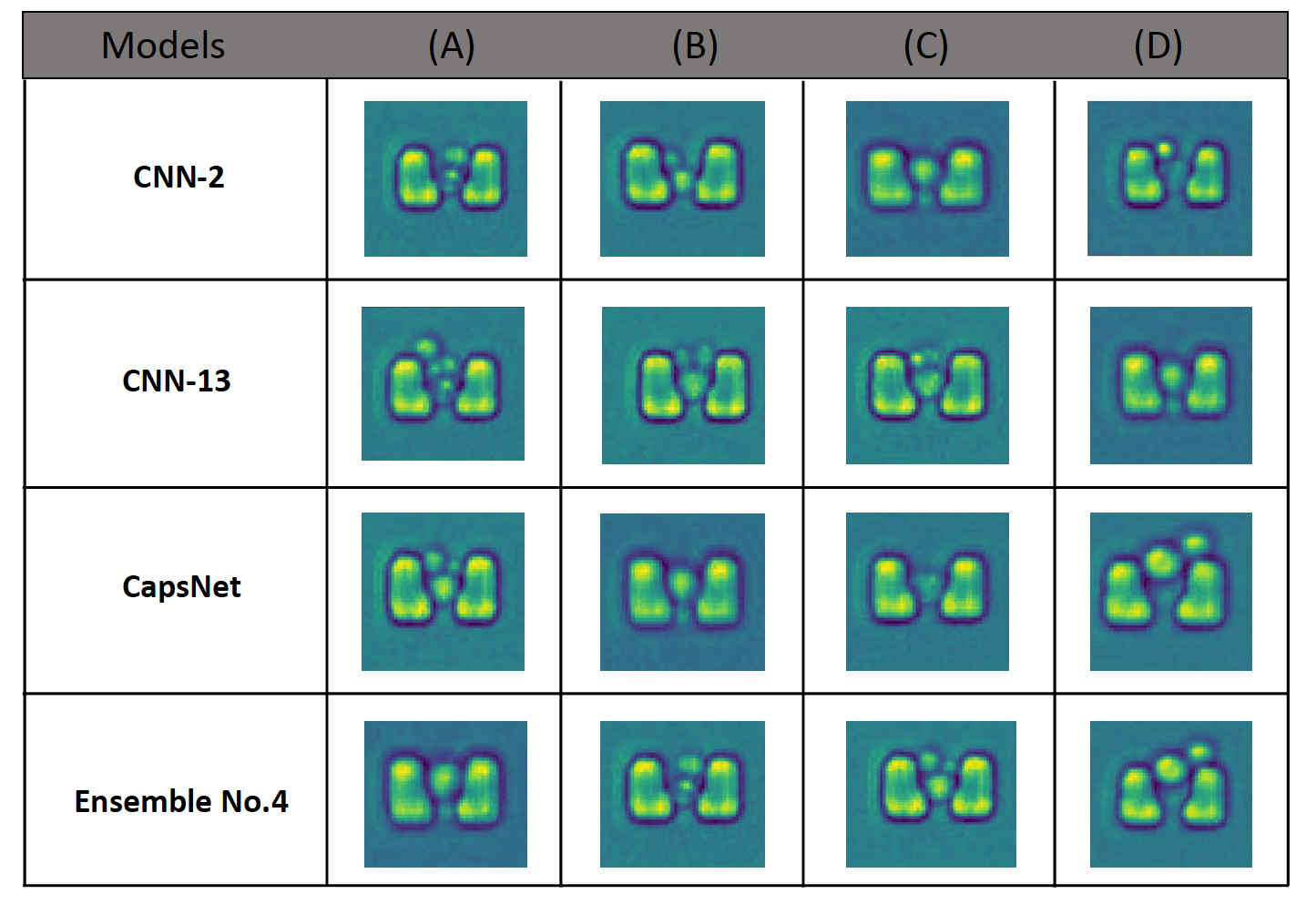

Supplement: S5 Table — CNN-2 (A) label exC: prediction mduC, CNN-2 (B) label mduC: prediction mC, CNN-2 (C) label mddC: prediction mC, CNN-2 (D) label mduC: predicted mddC. CNN-13 (A) label exC: prediction mduC, CNN-13 (B) label exC: prediction mduC, CNN-13 (C) label exC: prediction mduC, CNN-13 (D) label mddC: prediction mC. CapsNet (A) label exC: prediction mduC, CapsNet (B) label mddC: prediction mC, CapsNet (C) label mC: prediction mduC, CapsNet (D) label mduC: prediction exC. Ensemble No.4 (A) label mdC: prediction mC, Ensemble No.4 (B) label exC: prediction mdC, Ensemble No.4 (C) label exC: prediction mdC, Ensemble No.4 (D) label mdC: prediction exC. (TIF) [file pone.0246988.s008.tif]
